# *QuickStats: *Age-Adjusted Rate* for Suicide,^†^ by Sex — National Vital Statistics System, United States, 1975–2015

**DOI:** 10.15585/mmwr.mm6610a7

**Published:** 2017-03-17

**Figure:**
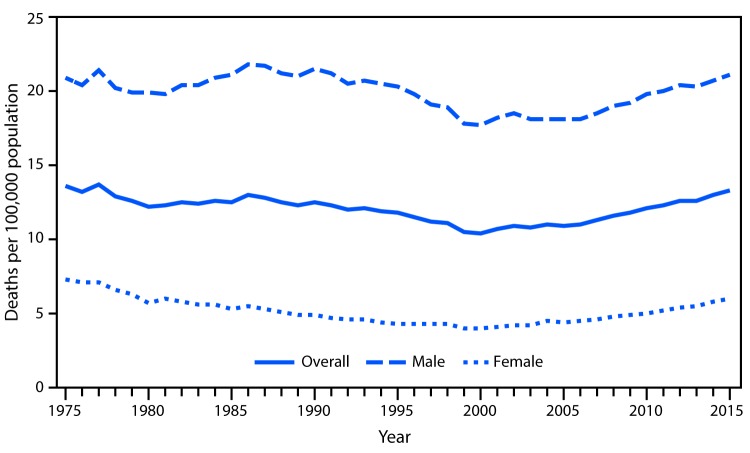
There was an overall decline of 24% in the age-adjusted suicide rate from 1977 (13.7 per 100,000) to 2000 (10.4). The rate increased in most years from 2000 to 2015. The 2015  suicide rate (13.3) was 28% higher than in 2000. The rates for males and females  followed the overall pattern; however, the rate for males was approximately 3–5 times higher than the rate for females throughout the study period.

**Source:** CDC. National Vital Statistics System. Mortality data. https://www.cdc.gov/nchs/nvss/deaths.htm.

For more information on this topic, CDC recommends the following link: https://www.cdc.gov/violenceprevention/suicide/index.html.

